# Stability of barotropic vortex strip on a rotating sphere

**DOI:** 10.1098/rspa.2017.0883

**Published:** 2018-02-28

**Authors:** Sung-Ik Sohn, Takashi Sakajo, Sun-Chul Kim

**Affiliations:** 1 Department of Mathematics, Gangneung-Wonju National University, Gangneung 25457, Korea; 2 Department of Mathematics, Kyoto University, Kyoto 606-8502, Japan; 3 Department of Mathematics, Chung-Ang University, Seoul 06974, Korea

**Keywords:** barotropic flow, vortex dynamics, rotating sphere, contour dynamics, linear stability

## Abstract

We study the stability of a barotropic vortex strip on a rotating sphere, as a simple model of jet streams. The flow is approximated by a piecewise-continuous vorticity distribution by zonal bands of uniform vorticity. The linear stability analysis shows that the vortex strip becomes stable as the strip widens or the rotation speed increases. When the vorticity constants in the upper and the lower regions of the vortex strip have the same positive value, the inner flow region of the vortex strip becomes the most unstable. However, when the upper and the lower vorticity constants in the polar regions have different signs, a complex pattern of instability is found, depending on the wavenumber of perturbations, and interestingly, a boundary far away from the vortex strip can be unstable. We also compute the nonlinear evolution of the vortex strip on the rotating sphere and compare with the linear stability analysis. When the width of the vortex strip is small, we observe a good agreement in the growth rate of perturbation at an early time, and the eigenvector corresponding to the unstable eigenvalue coincides with the most unstable part of the flow. We demonstrate that a large structure of rolling-up vortex cores appears in the vortex strip after a long-time evolution. Furthermore, the geophysical relevance of the model to jet streams of Jupiter, Saturn and Earth is examined.

## 1. Introduction

Jet streams are the prominent flow structures observed in atmospheric flows on Earth and gas planets such as Jupiter & Saturn [1,2]. It is of fundamental significance to understand how these jet structures appear, persist for a long time and become unstable in the longitudinal direction. The polar jet stream on Earth often intrudes into mid-latitudes and affects cyclonic storms in the atmosphere [3], thus predicting their course has become an important part of weather forecasting [4]. In this regard, the stability of jets is highly important.

The first step to understanding the complex phenomena of atmospheric flows, theoretically, is by introducing a simple mathematical model using a limited number of physical quantities. One such model is the barotropic model which considers an incompressible fluid of uniform density on the surface of a sphere. A remarkable contribution of this model to the understanding of zonal jets was provided by Rhines [5], where he found that, under a certain circumstance, the rotational effect and the energy cascade give rise to anisotropic east–west zonal band flows with a characteristic width known as the *Rhines scale*. The first numerical attempt to reproduce the two-dimensional turbulent flows with zonal jets in a plane with a rotational effect was made by Williams [6] using the barotropic model subject to random forcing. Subsequent numerical investigations have verified his numerical result in the case of a rotating sphere. Yoden & Yamada [7] have shown that the dynamics of unforced barotropic turbulence leads to a flow associated with a big circumpolar vorticity region, not zonal jets, as its quasi-final state. By using a homogeneous random forcing, Nozawa & Yoden [8] found that the barotropic flow generates, as a quasi-static two-dimensional turbulent state, strong circumpolar vortices accompanied by weak zonal jets when the rotation speed is moderate. Huang & Robinson [9] also constructed persistent latitudinal jet structures. Later, further numerical computation of the two-dimensional forced turbulence [10] showed that those zonal jets merge after a long-time evolution, and only a small number of zonal jets can survive as its asymptotic state. Note that other mathematical models such as shallow water models, general circulation models, quasi-geostrophic models and deep convection models have been used for further studies of jet streams on planets [1,11,12].

In view of the geophysical importance of zonal jets, we study the evolution of a latitudinal band region of a constant vorticity, called *a vortex strip* and investigate its stability. The linear stability of zonal jets on a β-plane has been studied by Obuse *et al.* [13]. They showed that these zonal jets are linearly unstable, and their instability triggers the merger of jets after a long-time evolution. However, the stability of zonal jets on a rotating sphere has not yet been investigated in detail so far. Our model for a vortex strip is based on the barotropic flow of an incompressible inviscid fluid on a rotating sphere. The linear stability and nonlinear evolutions of a vortex strip and a polar vortex cap on a non-rotating sphere were presented by Dritschel & Polvani [14,15]. In this sense, the purpose of this study is to extend their results to the case of a rotating sphere.

Here, we apply the vortex contour dynamics [16,17] to study the linear stability and to compute the nonlinear evolution of a vortex strip. A continuous vorticity distribution by the effect of the solid body rotation is approximated by a set of zonal band regions with piecewise constant vorticity. Embedding the vortex structure into these background vortex bands, we consider their evolution by tracking the boundaries of the vortex strip and the vortex bands. This approach has been used successfully in previous studies. For instance, the motion of a pair of vortex points [18] and a ring structure of *N* point vortices [19] on a rotating sphere were investigated through this approach.

The vortex strip can be regarded as a simple model of the shear layer, and several authors have presented numerical simulations of it. The evolution of a vortex strip in the plane, in connection with the Kelvin–Helmholtz instability, was studied by Pozrikidis & Higdon [20] and Roberts & Christiansen [21]. They found that the strip rolls up into a chain of vortices connected by so-called, *braids*. Recently, Bosler *et al.* [22] and Xiao *et al.* [23] reported numerical results for a thin vortex strip and zonal jet on a rotating sphere by using a Lagrangian particle/panel method and the RBF-vortex method, respectively. Most of these studies considered only thin vortex strips and did not examine thoroughly the dependence of the evolution of the strip on the physical parameters.

Our main concern in this paper is the investigation of the stability of a vortex strip, i.e. unstable modes, growth rates and dependence on the rotation speed and the width of the strip, etc. Our aim is not the reproduction of the numerical results mentioned above. The numerical computation for vortex strips is conducted only up to the moderately nonlinear stage, but for a wide range of parameters, to compare with the linear stability. We thus rely on the framework of the vortex contour dynamics for the nonlinear evolution. We will therefore not be using advanced numerical methods such as the spectral method [24] and the semi-Lagrangian or Lagrangian methods [22,23,25,26]. The vortex contour dynamics is efficient to accomplish our purpose, and provides an advantage over the spectral method because the stability of the vortex strip is described not as the growth of spectra of its global spherical harmonic representation, but rather, in terms of the local deformation of contour curves. Owing to this property, we can examine not only whether the zonal jets are unstable, but also which wavenumber of local perturbations becomes the most unstable. Additionally, it allows us to provide a detailed description of how the instability of local zonal jets affects the flow of the whole sphere.

This paper is organized as follows. In §2, we describe the flow geometry of the vortex strip on a rotating sphere and explain how to approximate the flow in the framework of vortex contour dynamics. In §3, we present the linear stability analysis for vortex strips. Numerical computations of the nonlinear evolution of vortex strips are given in §4. The geophysical relevance of the vortex strip to the jet streams of Jupiter, Saturn and Earth is also discussed in §5. In the final section, we present our conclusion.

## 2. Flow descriptions

### (a) Distributions of vorticity

We consider a simple geophysical fluid model, the so-called *barotropic model*, in which the fluid is inviscid and incompressible, and of constant density on the surface of the unit sphere. The vorticity *ω* and the stream function *ψ* satisfy

2.1 $$\omega =\nabla_{\Sigma }^{2}\psi =\frac{1}{\cos\theta }\frac{\partial }{\partial \theta }\left(\cos\theta \frac{\partial \psi }{\partial \theta }\right)+\frac{1}{\cos^{2}\theta }\frac{\partial^{2}\psi }{\partial \varphi^{2}}.$$

Here, $\nabla_{\Sigma }^{2}$ denotes the Laplace–Beltrami operator on the sphere in spherical coordinates (*θ*,*φ*)∈[−*π*/2,*π*/2]×[0,2*π*). The zonal and the meridional velocities, *u* and *v*, are derived from the stream function via the following geostrophic relation:

2.2 $$u(\theta ,\varphi )=-\frac{\partial \psi }{\partial \theta }\;\mathrm{and}\;v(\theta ,\varphi )=\frac{1}{\cos\theta }\frac{\partial \psi }{\partial \varphi }.$$

Let us describe the basic flow of a vortex strip on a rotating sphere considered in this paper. Suppose that the boundaries of a vortex strip are located between two latitudinal lines *θ*=*θ*_*b*_1__, *θ*_*b*_2__ on the sphere and that the vorticity constants are given by *ω*_*N*_ for *θ*_*b*_1__<*θ*<*π*/2, *ω*_*b*_ for *θ*_*b*_2__<*θ*<*θ*_*b*_1__ and *ω*_*S*_ for −*π*/2<*θ*<*θ*_*b*_2__. As the vorticity distribution induced by the solid body rotation of the sphere becomes f(θ,φ)=2Ωsin⁡θ, where *Ω* is its angular velocity, the absolute vorticity of a barotropic vortex strip is then represented by

2.3 $$\omega (\theta ,\varphi )=\left\{ \begin{array}{ll} \omega_{N}+2\Omega \;\sin\theta & \text{for}\;\theta_{b_{1}}<\theta <\frac{\pi }{2},\\ \omega_{b}+2\Omega \;\sin\theta & \text{for}\;\theta_{b_{2}}<\theta <\theta_{b_{1}},\\ \omega_{S}+2\Omega \;\sin\theta & \text{for}\;-\frac{\pi }{2}<\theta <\theta_{b_{2}}. \end{array}\right.$$

**Figure 1.**
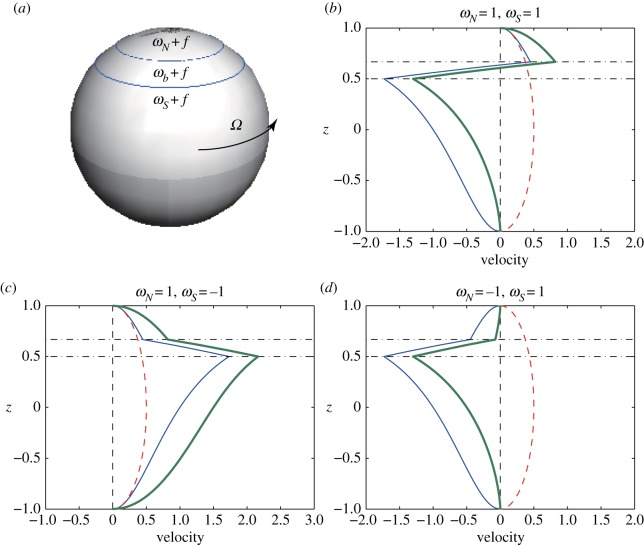
(*a*) Schematic of the vorticity distribution of a vortex strip on a rotating sphere. (*b*–*d*) Profiles of the zonal velocity. The vorticity constants are given by (*b*) (*ω*_*N*_,*ω*_*S*_)=(1,1), (*c*) (*ω*_*N*_,*ω*_*S*_)=(1,−1) and (*d*) (*ω*_*N*_,*ω*_*S*_)=(−1,1). The thick (green) curves represent the velocity fields induced by the vortex strip (2.3) between *z*_*b*_1__=2/3 and *z*_*b*_2__=1/2 on the rotating sphere with the angular speed *Ω*=0.5. The thin (blue) curves and the dotted (red) curves represent the velocity fields induced only by the zonal strip, i.e. (2.3) with *Ω*=0, and by the solid body rotation, respectively.

A schematic of the vorticity distribution is shown in figure 1*a*. Owing to Gauss’s theorem, the total vorticity over the sphere $S^{2}$ should be zero. As the integration of the vorticity induced by the solid body rotation is zero, i.e. $\int_{S^{2}}2\Omega \;\sin\theta \;dA=0$, we may consider only the integration of the constant vorticities. Hence, the vorticity constants, *ω*_*N*_, *ω*_*b*_ and *ω*_*S*_ satisfy

2.4 $$(1-z_{b_{1}})\omega_{N}+(z_{b_{1}}-z_{b_{2}})\omega_{b}+(1+z_{b_{2}})\omega_{S}=0,$$

where $z_{b_{1}}=\sin\theta_{b_{1}}$ and $z_{b_{2}}=\sin\theta_{b_{2}}$. The constraint (2.4) is the same as that for the sphere without rotation considered by Dritschel & Polvani [14].

We examine relation (2.4) among the vorticity constants and the profile of the velocity induced by the barotropic vortex strip. If we assume that the vorticity constants of the northern and the southern polar regions are equal, say *ω*_*N*_=*ω*_*S*_=1, then equation (2.4) yields

2.5 $$\omega_{b}=1-\frac{2}{z_{b_{1}}-z_{b_{2}}},$$

which is always negative owing to −1<*z*_*b*_2__<*z*_*b*_1__<1 and diverges monotonically as the width of the vortex strip *h*:=*z*_*b*_1__−*z*_*b*_2__ tends to zero. The zonal velocity profile induced by the barotropic vortex strip (2.3) between *z*_*b*_1__=2/3 and *z*_*b*_2__=1/2 on the rotating sphere with the angular speed *Ω*=0.5 is shown by the thick (green) curve in figure 1*b*. In the same figure, as a reference, the velocity fields induced by the piecewise constant vorticity for the vortex strip and by the vorticity distribution f(θ,φ)=2Ωsin⁡θ of the solid body rotation are shown as the thin (blue) curve and the dotted (red) curve, respectively. The thin curve indicates that a strong shear is formed owing to the presence of the vortex strip, which results in the change of the zonal velocity from the eastward direction, above the strip, to the westward direction, below the strip. The dotted curve demonstrates that the solid body rotation gives rise to an eastward zonal flow. When those two flows are combined, as shown by the thick curve, we still observe the strong shear flow inverting the flow direction through the vortex strip.

Suppose next that *ω*_*N*_=1 and *ω*_*S*_=−1. Then from equation (2.4), it follows

2.6 $$\omega_{b}=\frac{z_{b_{1}}+z_{b_{2}}}{z_{b_{1}}-z_{b_{2}}}.$$

When the vortex strip is located in the northern hemisphere, i.e. 0<*z*_*b*_2__<*z*_*b*_1__<1, *ω*_*b*_ is well defined and positive for any *z*_*b*_1__ and *z*_*b*_2__ owing to *z*_*b*_1__+*z*_*b*_2__≠0. Hence, it is uniquely expressed as *ω*_*b*_(*r*)=(1+*r*)/(1−*r*) for 0<*r*=*z*_*b*_2__/*z*_*b*_1__<1, which indicates that $0<\omega_{b}<\infty$ is monotone increasing with $\lim_{r\to 0}\omega_{b}(r)=0$ and $\lim_{r\to 1}\omega_{b}(r)=\infty$. In other words, *ω*_*b*_ is always positive and diverges as the width of the vortex strip tends to zero. Similarly, for the vortex strip in the southern hemisphere, −1<*z*_*b*_2__<*z*_*b*_1__<0, we have $-\infty <\omega_{b}(r)<0$ for *r*>1, which means that *ω*_*b*_ is always negative and diverges as the width vanishes. For −1<*z*_*b*_2__<0<*z*_*b*_1__<1, *ω*_*b*_ has a value between −1 and 1. Special care should be taken in the limiting cases where $z_{b_{1}}\to 0+$ and $z_{b_{2}}\to 0-$. As *ω*_*b*_(*r*)=(1+*r*)/(1−*r*) for *r*<0, *ω*_*b*_ can take any value in (−1,1) in the zero limit of *z*_*b*_2__=*rz*_*b*_1__. Additionally, when *z*_*b*_1__ is zero, *ω*_*b*_=*ω*_*S*_=−1 holds regardless of the location of the lower boundary *z*_*b*_2__, which results in no difference in the vorticity constant across the lower boundary. The same situation occurs for *ω*_*b*_=*ω*_*N*_=1 when *z*_*b*_2__=0. Therefore, we exclude a consideration of the vortex strip in the case of *z*_*b*_1__=0 or *z*_*b*_2__=0 when *ω*_*N*_*ω*_*S*_=−1. Figure 1*c* shows the velocity profile of the vortex strip between *z*_*b*_1__=2/3 and *z*_*b*_2__=1/2 for *ω*_*N*_=1 and *ω*_*S*_=−1 with *Ω*=0.5. All the velocity fields are positive and the solid body rotation enhances the eastward velocity of the vortex strip.

Finally, when *ω*_*N*_=−1 and *ω*_*S*_=1, *ω*_*b*_ is given by *ω*_*b*_=−(*z*_*b*_1__+*z*_*b*_2__)/(*z*_*b*_1__−*z*_*b*_2__), which means that *ω*_*b*_ is positive and diverges as the width goes to zero. A similar situation arises in the limiting case when $z_{b_{1}}\to 0+$ and $z_{b_{2}}\to 0-$ as that for *ω*_*N*_=1 and *ω*_*S*_=−1. Figure 1*d* shows the velocity profile of the vortex strip for *ω*_*N*_=−1 and *ω*_*S*_=1 with *Ω*=0.5. The velocity profile corresponding to the piecewise constant vorticity field has the opposite direction to the velocity induced by the solid body rotation.

### (b) Contour dynamics formulation

We approximate the continuous vorticity distribution induced by the solid body rotation of the sphere with the *M*+1 zonal bands with piecewise-constant vorticity separated by *M* latitudinal boundaries. To track the evolution of those boundaries, the flow on the surface of a sphere is embedded into three-dimensional space, using the three-dimensional Cartesian representation of those boundaries as described in [18,19]. We here comment on a different approach by Surana & Crowdy [27] which maps the boundaries to the complex plane through the stereographic projection and considers the analytic representation of the flows. This approach may be extended to the flow of a rotating sphere, which will be discussed later.

Let **ℓ**_*k*_(*α*,*t*), *k*=1,…,*M* denote the boundary curves where *α* is the Lagrangian parameter along the curve and *t* is the time. Then the evolution equation of the curve **ℓ**_*k*_(*α*,*t*) is described by the following boundary integrals with respect to the *M* boundaries:

2.7 $$\frac{\partial \ell_{k}}{\partial t}(\alpha ,t)=-\frac{1}{2\pi }\sum_{m=1}^{M}\omega_{m}^{\prime }\int_{0}^{2\pi }\log|\ell_{k}(\alpha ,t)-\ell_{m}(\alpha ,t)|\frac{\partial \ell_{m}}{\partial \alpha }(\alpha ,t)\;\text{d}\alpha ,$$

where $\omega_{m}^{\prime }$ represents the vorticity jump between the boundaries **ℓ**_*m*−1_(*α*,*t*) and **ℓ**_*m*_(*α*,*t*) for *m*=1,…,*M*. Note that **ℓ**_0_ and **ℓ**_*M*+1_ are regarded as the north and south poles, respectively. The initial configuration is given by

2.8 $$\ell_{k}(\alpha ,0)=(\sqrt{1-z_{k0}^{2}}\cos\alpha ,\sqrt{1-z_{k0}^{2}}\sin\alpha ,z_{k0}),$$

with

2.9 $$z_{k0}=1-\frac{2k}{M+1}.$$

Let us note that each band with (2.9) has the same area because of the same height. The vorticity jump constant $\omega_{k}^{\prime }$ of the *k*th band is chosen to be the average of vorticity at the band boundaries in the piecewise constant vorticity. For numerical convenience, we assume here that the two boundaries of a zonal vortex strip coincide with some of the *M* latitudinal band boundaries approximating the solid body rotation at the initial moment. Therefore, for the vortex strip, the vorticity constant *ω*_*k*_ for each vortex band 1≤*k*≤*M*+1 is given by

2.10 $$\omega_{k}=\left\{ \begin{array}{ll} \Omega (z_{0,k-1}+z_{0,k})+\omega_{N} & \text{for}\;1\leq k<k_{b_{1}},\\ \Omega (z_{0,k-1}+z_{0,k})+\omega_{b} & \text{for}\;k_{b_{1}}<k<k_{b_{2}},\\ \Omega (z_{0,k-1}+z_{0,k})+\omega_{S} & \text{for}\;k_{b_{2}}<k\leq M+1, \end{array}\right.$$

where *k*_*b*_1__,*k*_*b*_2__∈{1,…,*M*} are the indices of the band boundaries of the vortex strip. The vorticity jump $\omega_{k}^{\prime }$ is then specified by

2.11 $$\omega_{k}^{\prime }=\left\{ \begin{array}{ll} \Omega (z_{0,k-1}-z_{0,k+1}) & \text{for}\;k\neq k_{b_{i}},\\ \Omega (z_{0,k-1}-z_{0,k+1})+\omega_{N}-\omega_{b} & \text{for}\;k=k_{b_{1}},\\ \Omega (z_{0,k-1}-z_{0,k+1})+\omega_{b}-\omega_{S} & \text{for}\;k=k_{b_{2}}, \end{array}\right.$$

in which the vortex strip is embedded in the region between the two band boundaries *z*=*z*_*k*_*b*_1___ and *z*=*z*_*k*_*b*_2___ with 1≤*k*_*b*_1__<*k*_*b*_2__≤*M*.

**Figure 2.**
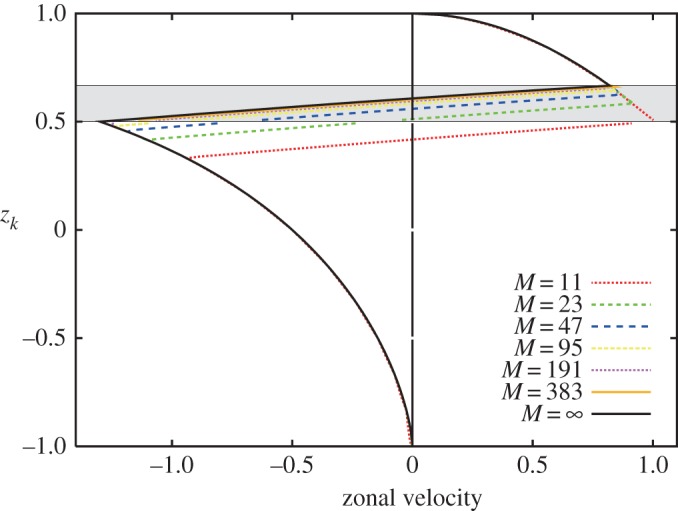
The zonal velocity profiles induced by the vortex strip (2.3) between *z*_*b*_1__=2/3 and *z*_*b*_2__=1/2, which is represented by a band region filled with grey, on the rotating sphere with the angular speed *Ω*=0.5. The flow is approximated by *M* zonal band boundaries from *M*=11 to *M*=383.

We check how many boundaries are required to approximate the continuous vorticity distribution corresponding to the solid body rotation. Figure 2 shows the zonal velocity profiles induced by the vorticity strip (2.3) between *z*_*b*_1__=2/3 and *z*_*b*_2__=1/2 on the rotating sphere with *Ω*=0.5 approximated by *M*=11, 23, 47, 95, 191 and 373 band boundaries. While we observe a clear convergence with respect to *M*, the velocity field is poorly approximated for *M*=12, 23 and 47 in the sense that the boundaries of the strong shear differ significantly from the boundaries of the vortex strip. Accordingly, we hereafter take *M*=95 as the number of the vortex band boundaries in the following stability analysis and numerical computations.

## 3. Linear stability analysis

Let us explain the meaning of the stability of flows in our study. The zonal band configuration *z*_*k*0_ in (2.9) represents a steady state of equation (2.7). We consider the linear stability of the boundaries **ℓ**_*k*_(*α*,0) for *k*=1,…,*M*, giving an infinitesimal perturbation proportional to exp⁡[i(mα−σt)] in the latitudinal direction. The temporal growth rate *σ* depends not only on *Ω* and *m*, but also on (*θ*_*b*_1__,*θ*_*b*_2__) for the vortex strip. We mainly pay attention to how the growth rate for a given mode *m*, i.e. the imaginary part of *σ*, is affected by the angular speed of rotation *Ω* and the width of the vortex strip $h:=\sin\theta_{b_{1}}-\sin\theta_{b_{2}}$.

Let us denote the *k*th boundary as

3.1 $$z_{k}(\alpha ,t)=z_{k0}+\zeta_{k}(\alpha ,t),\;k=1,\ldots ,M,$$

where *ζ*_*k*_ represents the perturbation added to the *k*th boundary. Substituting it into the *z*-component of (2.7) and linearizing it, we obtain

3.2 $$\frac{\text{d}\zeta_{k}}{\text{d}t}=\frac{\partial \zeta_{k}}{\partial t}+\Omega_{k}\frac{\partial \zeta_{k}}{\partial \alpha }=-\frac{1}{2\pi }\sum_{m=1}^{M}\omega_{m}^{\prime }\int_{0}^{2\pi }\log|\ell_{k}(\alpha ,t)-\ell_{m}(\alpha ,t)|\frac{\partial \zeta_{m}}{\partial \alpha }(\alpha ,t)\;\text{d}\alpha ,$$

where

3.3 $$\Omega_{k}=\left\{ \begin{array}{ll} \Omega +\frac{\omega_{N}}{1+z_{k}} & \text{for}\;1\leq k\leq k_{b_{1}},\\ \Omega +\frac{1}{1-z_{k}^{2}}[\omega_{b}(z_{b_{1}}-z_{k})+\omega_{N}(1-z_{b_{1}})] & \text{for}\;k_{b_{1}}<k<k_{b_{2}},\\ \Omega -\frac{\omega_{S}}{1-z_{k}} & \text{for}\;k_{b_{2}}\leq k\leq M \end{array}\right.$$

is the corresponding angular velocity. For each *k*, the value of *Ω*_*k*_ is determined from the direct calculation of the steady configuration of the band boundaries (2.9). For example, as equation (2.1) is written as

3.4 $$-\frac{d}{dz}\left[{\left(1-z^{2}\right)}^{1/2}u\right]=\omega ,$$

we have *Ω*_*k*_*b*_1__+1_ for the zonal flow:

$$\Omega_{k_{b_{1}}+1}=\frac{1}{1-z_{k_{b_{1}+1}}^{2}}\left\{\int_{z_{k_{b_{1}}+1}}^{z_{k_{b_{1}}}}\omega \;\text{d}z+\int_{z_{k_{b_{1}}}}^{1}\omega \;\text{d}z\right\}=\Omega -\frac{(z_{k_{b_{1}}}-z_{k_{b_{1}}+1})\omega_{b}+(1-z_{k_{b_{1}}})\omega_{N}}{1-z_{k_{b_{1}}+1}^{2}}.$$

The third equation of (3.3) can be found by using the constraint (2.4).

Let us set the perturbation vector ***ζ***=(*ζ*_1_,…,*ζ*_*M*_) as

3.5 $$\zeta =\hat{\zeta }\;e^{i(m\alpha -\sigma^{\left(m\right)}t)}+\text{c.c.}=2\;\text{Re}[\hat{\zeta }\;{\text{e}}^{i(m\alpha -\sigma^{\left(m\right)}t)}],$$

where $\hat{\zeta }=({\hat{\zeta }}_{1},\ldots ,{\hat{\zeta }}_{M})$ denotes the Fourier amplitude vector of the perturbation for a given longitudinal wavenumber *m*, and c.c. represents its complex conjugate. Then, we obtain the eigenvalue problem for the amplitude vector,

3.6 $$(A-\sigma^{\left(m\right)}I)\hat{\zeta }=0.$$

Here, *A* is an *M*×*M* matrix with the complex components

3.7 $$A_{jk}=-\frac{\omega_{k}^{\prime }}{2}F_{m}(z_{0j},z_{0k})+\delta_{jk}m\Omega_{k},\;F_{m}(z_{a},z_{b})={\left(\frac{1-z_{>}}{1+z_{>}}\frac{1+z_{<}}{1-z_{<}}\right)}^{m/2},$$

where $z_{>}=\max(z_{a},z_{b})$ and $z_{<}=\min(z_{a},z_{b})$. The expression of *A* is analogous to that given by Dritschel & Polvani [18]. Solving the eigenvalue problem, we obtain *M* eigenvalues, which are denoted as $\sigma_{k}^{\left(m\right)}$ for *k*=1,…,*M*.

We take the vorticity constants *ω*_*N*_=*ω*_*S*_=1 and use *M*+1 vortex band boundaries with *M*=95 to approximate the solid body rotation. We examine the cases when the vortex strip is located between two band boundaries with even indices, namely *k*_*b*_1__=8*p* and *k*_*b*_2__=8*q* with 1≤*p*<*q*≤11. The stability of the flow is numerically confirmed as follows. First, we calculate the eigenvalues $\sigma_{k}^{\left(m\right)}$, *k*=1,…,*M* for almost all modes 2≤*m*<100. If the growth rates are all zero, i.e. $\text{Im}[\sigma_{k}^{\left(m\right)}]=0$ for all *k*=1,…,*M* and 2≤*m*<100 up to a numerical tolerance, then the flow is regarded to be neutrally stable. However, the flow is linearly unstable if there exists an eigenvalue with a positive imaginary part. As a matter of fact, in all the numerical results shown below, there exist only two eigenvalues with non-zero imaginary parts for each *m* when the flow is not linearly stable; one has a positive imaginary part and the other has a negative imaginary part with the same magnitude. We here note that the existence of the unstable eigenvalue with a positive growth rate means that not only the boundaries of the vortex strip but also all band boundaries become unstable as a whole. This will be discussed in more details shortly.

For a given pair of even (*k*_*b*_1__,*k*_*b*_2__) with *k*_*b*_1__<*k*_*b*_2__, we calculate the eigenvalues for modes 2≤*m*<100. As we mentioned above, if the flow is linearly unstable, there exists only one eigenvalue with its imaginary part being positive. Accordingly, for notational convenience, we assume that $\sigma_{1}^{\left(m\right)}$ denotes the unstable eigenvalue $\text{Im}[\sigma_{1}^{\left(m\right)}]>0$ and the other $\sigma_{k}^{\left(m\right)}$ satisfy $\text{Im}[\sigma_{k}^{\left(m\right)}]\leq 0$ for *k*=2,…,*M*. We then define the most unstable mode by

3.8 $$m_{\max}=\underset{2\leq m<100}{\text{argmax}}\text{Im}[\sigma_{1}^{\left(m\right)}].$$

**Figure 3.**
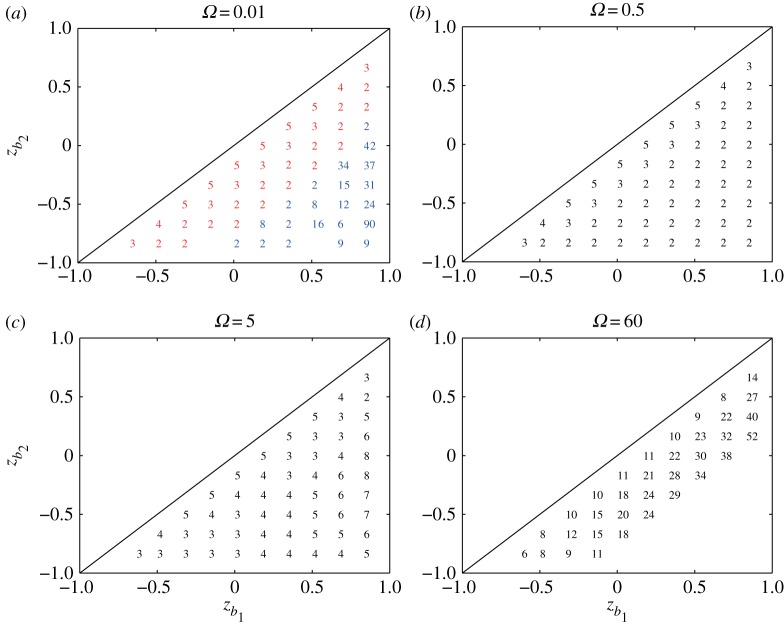
Stability diagrams showing the most unstable mode *m*_*max*_ for the vortex strip whose boundaries are located at *z*_*k*_*b*_1___ and *z*_*k*_*b*_2___ on the rotating sphere with the angular speed (*a*) *Ω*=0.01, (*b*) *Ω*=0.5, (*c*) *Ω*=5 and (*d*) *Ω*=60. The continuous vorticity distribution corresponding to the solid body rotation is approximated by *M*=95 band boundaries. The vorticity constants are given by *ω*_*N*_=*ω*_*S*_=1. In (*a*), the modes coloured with blue correspond to small associated growth rates and the modes in the remaining region are coloured with red.

**Figure 4.**
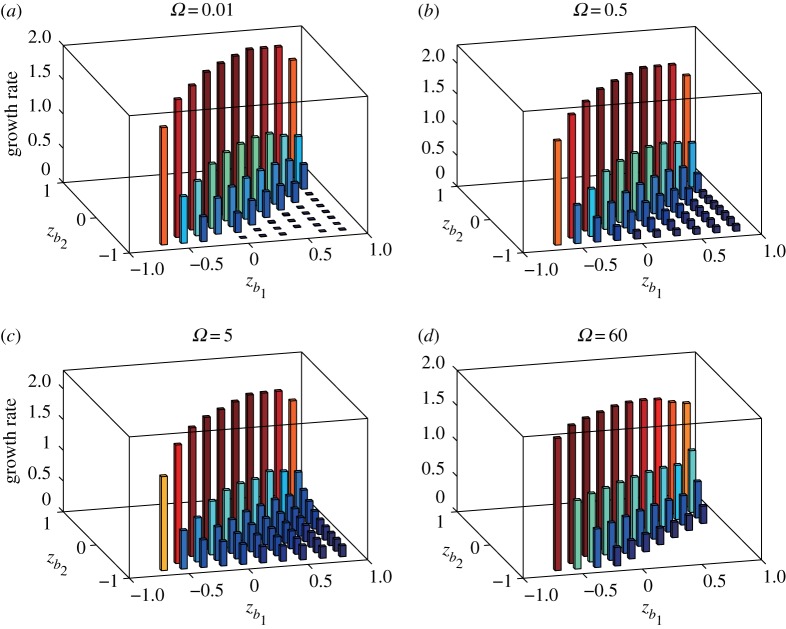
Plots of the growth rates corresponding to the most unstable mode *m*_*max*_ in figure 3 for the flow of the vortex strip on the rotating sphere with *Ω*=0.01,0.5,5 and 60. The number of the discretizing vortex band boundaries is *M*=95. The vorticity constants are given by *ω*_*N*_=*ω*_*S*_=1.

A stability diagram is the plot of the unstable modes *m*_*max*_ for various locations of the vortex strip between *z*=*z*_*k*_*b*_1___ and *z*=*z*_*k*_*b*_2___. Figure 3 shows the stability diagrams for *Ω*=0.01,0.5,5 and 60. When the imaginary parts of the eigenvalues are zero for all *m*<100, i.e. the flow is neutrally stable, no number is displayed. For *Ω*≤5, we find in figure 3*a*–*c* that the flow is unstable for almost all pairs of (*k*_*b*_1__,*k*_*b*_2__). For *Ω*=60, the unstable region shrinks greatly as we see in figure 3*d*. Therefore, the rotation of the sphere gives a stabilizing effect on the flow with the vortex strip, which will be examined later. When we observe the stability diagram for *Ω*=0.5 more closely, the most unstable mode gets larger as *z*_*k*_*b*_2___ approaches the diagonal line along a fixed *z*_*k*_*b*_1___. This behaviour is similar to the result found by Dritschel & Polvani [14] for the non-rotating case. As the rotation speed *Ω* increases, the monotone behaviour of the most unstable mode along a fixed *z*_*k*_*b*_1___ is broken and higher modes appear in the stability diagram. For *Ω*=60, the most unstable modes are observed in a small band region below the diagonal line *z*_*k*_*b*_1___=*z*_*k*_*b*_2___. The growth rate corresponding to the most unstable mode *m*_*max*_ in figure 3 is plotted in figure 4. We observe that, for fixed *z*_*k*_*b*_1___, the growth rate below the diagonal line becomes the largest and it decreases monotonically as *z*_*k*_*b*_2___ goes southwards. This indicates that the flow tends to be less unstable as the width *h* increases. When we pay attention to the growth rates along the diagonal line, the peak of the growth rate is attained at the equator for all the cases of *Ω*. Note also that the small growth rates in the right low region in figure 4*b* for *Ω*=0.5 increase slightly in figure 4*c* for *Ω*=5.

Figures 3*a* and 4*a* show the stability diagram and the associated growth rates for a small rotation speed *Ω*=0.01, which is taken to see the behaviour in the vanishing limit of *Ω*. The growth rates in the right lower region are very small, which implies a convergence to 0 and the vortex strip thus becomes stable as Ω→0. In the stability diagram, the modes in the lower right region correspond to small growth rates and are coloured with blue, and the modes in the remaining region are coloured with red. The shape of the red (unstable) region and the behaviour of the mode increasing along a fixed *z*_*k*_*b*_1___ are similar to those of the non-rotating sphere [14]. Furthermore, from figure 4*a*, we find that, for *Ω*>0, not too large, the strip of any width is unstable, whereas the strip with a large width is stable in the non-rotating case. Therefore, we conclude that the rotation produces an instability on the wide vortex strip when the rotation speed is small.

The eigenvector for the linearized equation gives rise to the unstable flow profile. Suppose that $X_{1}^{\left(m\right)}=x_{1}^{\left(m\right)}+iy_{1}^{\left(m\right)}$ denotes the eigenvector corresponding to the unstable eigenvalue $\sigma_{1}^{\left(m\right)}=a_{1}^{\left(m\right)}+ib_{1}^{\left(m\right)}$ for a given *m*, where $x_{1}^{\left(m\right)}$ and $y_{1}^{\left(m\right)}$ are *M*-dimensional real vectors. Then, the ansatz (3.5) yields the unstable perturbation vector $\zeta_{1}^{\left(m\right)}(\alpha ,t)$ for the band boundaries with respect to the mode *m*, which is expressed as

$$\zeta_{1}^{\left(m\right)}(\alpha ,t)=2\;\text{Re}[X_{1}^{\left(m\right)}\;{\text{e}}^{i(m\alpha -\sigma_{1}^{\left(m\right)}t)}]={\text{e}}^{b_{1}^{\left(m\right)}t}(x_{1}^{\left(m\right)}\;\cos(m\alpha -a_{1}^{\left(m\right)}t)-y_{1}^{\left(m\right)}\;\sin(m\alpha -a_{1}^{\left(m\right)}t)).$$

**Figure 5.**
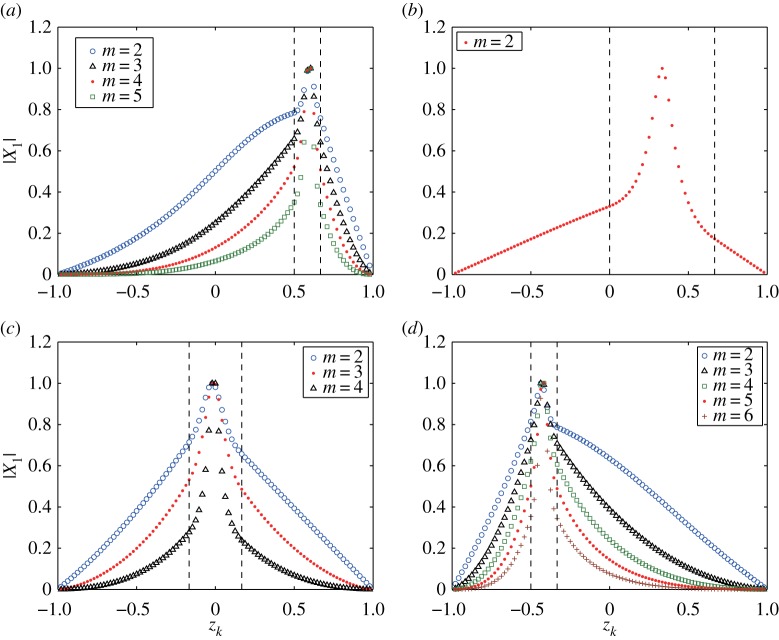
Distributions of the components of the eigenvectors $\left|X_{1}^{\left(m\right)}\right|$ corresponding to the unstable eigenvalues $\sigma_{1}^{\left(m\right)}$ for the modes 2≤*m*≤6. The boundary locations of the vortex strip, shown by the dashed vertical lines, are (*a*) (*k*_*b*_1__,*k*_*b*_2__)=(16,24), (*b*) (*k*_*b*_1__,*k*_*b*_2__)=(16,48), (*c*) (*k*_*b*_1__,*k*_*b*_2__)=(40,56) and (*d*) (*k*_*b*_1__,*k*_*b*_2__)=(64,72). The other parameters are given by *ω*_*N*_=*ω*_*S*_=1, *Ω*=0.5 and *M*=95.

As $b_{1}^{\left(m\right)}>0$, the perturbation in the $X_{1}^{\left(m\right)}$-direction grows exponentially. As examples, we choose the four cases (*k*_*b*_1__,*k*_*b*_2__)=(16,24), (16,48), (40,56) and (64,72) with *M*=95 and *Ω*=0.5 and compute the eigenvectors to the unstable eigenvalues. Figure 5 shows the distribution of the components of the eigenvectors associated with the unstable eigenvalues for the modes 2≤*m*≤6 as a function of *z*_*k*_∈(−1,1) for *k*=1,…,*M*. We normalize them by their magnitudes $\left|X_{1}^{\left(m\right)}\right|$ so that its largest component becomes one. In figure 5, the components of the eigenvector to the most unstable mode *m*_*max*_ are marked by the solid red circles, and some eigenvectors to the eigenvalues from the largest are also shown. For instance, in the case of (*k*_*b*_1__,*k*_*b*_2__)=(16,24), as the growth rates for modes 2≤*m*≤5 are $\sigma_{1}^{\left(2\right)}=1.249$, $\sigma_{1}^{\left(3\right)}=1.853$, $\sigma_{1}^{\left(4\right)}=2.008$ and $\sigma_{1}^{\left(5\right)}=1.585$, respectively, and the growth rates for *m*>6 are mostly zero, we plot the eigenvectors for *m*=2,3,4,5 in figure 5*a*. All the cases in figure 5 show that the distribution for each *m* has a peak in the middle of the lower and upper boundaries of the vortex strip.

The unstable eigenvector tells us how each boundary evolves under the initial perturbation. To describe it, let us recall a basic theory of differential equations. Suppose now that A is a matrix whose distinct eigenvalues and eigenvectors are denoted by *γ*_*k*_ with *γ*_*k*_≠*γ*_*j*_ (*k*≠*j*) and ***u***_*k*_ for *k*=1,…,*M*. Then a complex-valued solution $\hat{\xi }(t)\in C^{M}$ subject to the linear equation $(d/dt)\hat{\xi }=A\hat{\xi }$ with $\hat{\xi }(0)={\hat{\xi }}_{0}=\sum_{k=1}^{M}c_{k}u_{k}$ is expressed by $\hat{\xi }(t)=\sum_{k=1}^{M}c_{k}\exp(\gamma_{k}t)u_{k}$. In addition, if $\gamma_{1}\in C$ is the unstable eigenvalue with Im[*γ*_1_]>0 and the other *γ*_*k*_, *k*=2,…,*M* are either stable or neutrally stable eigenvalues satisfying Im[*γ*_*k*_]≤0, the solution evolves along the unstable direction as $\hat{\xi }(t)∼c_{1}\exp(\gamma_{1}t)u_{1}$. In our stability analysis, by solving the eigenvalue problem for a given mode *m*, we obtain *M* eigenvalues, $\sigma_{k}^{\left(m\right)}$ for *k*=1,…,*M*, with $\sigma_{1}^{\left(m\right)}$ being the unstable eigenvalue. Accordingly, if we give the initial perturbation, the unstable eigenvector $\left|X_{1}^{\left(m\right)}\right|$ suggests which boundary becomes more unstable than the others, because the disturbance grows with $\left|\zeta |=\exp(b_{1}^{\left(m\right)}t)|X_{1}^{\left(m\right)}\right|$. The unstable eigenvectors shown in figure 5 have strong peaks in the middle of the vortex strip. Therefore, when *ω*_*N*_=*ω*_*S*_=1, the inner flow of the vortex strip is the most unstable one for all the cases examined here.

**Figure 6.**
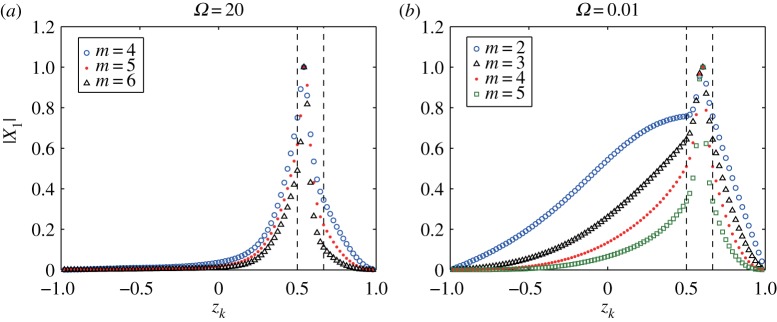
Distributions of the magnitude of the normalized eigenvectors $\left|X_{1}^{\left(m\right)}\right|$ associated with the unstable eigenvalues $\sigma_{1}^{\left(m\right)}$ for modes 2≤*m*≤5. The vortex strip is located between *z*_16_=2/3 and *z*_24_=1/2. The angular speeds are (*a*) *Ω*=20 and (*b*) *Ω*=0.01. The vorticity constants are given by *ω*_*N*_=*ω*_*S*_=1.

In order to discuss the limiting behaviour of the eigenvector in terms of the angular speed *Ω*, we choose two values, a large one *Ω*=20 and a very small one *Ω*=0.01. Figure 6 shows the distributions of the magnitude of the eigenvectors $\left|X_{1}^{\left(m\right)}\right|$ corresponding to the unstable eigenvalue $\sigma_{1}^{\left(m\right)}$ for 2≤*m*≤6. The vortex strip is located between *z*_16_=2/3 and *z*_24_=1/2. In figure 6*a* for *Ω*=20, the eigenvectors for *m*=2 and 3 are not shown, because their corresponding growth rates are small. The peak slightly moves to the lower boundary of the vortex strip at *z*_24_=1/2 for all the modes *m*. For *Ω*=0.01 in figure 6*b*, the peak of each distribution is located in the middle of the vortex strip, similar to the case of *Ω*=0.5. This figure shows that the distribution of the eigenvector in the limit as Ω→0 does not converge to that for the non-rotating sphere. Note that, in the non-rotating sphere, the eigenvector has only two components of the same magnitude at the two boundaries of the vortex strip, which means the most unstable boundaries. Consequently, these facts reflect a fundamental difference of the vortex strips in the rotating sphere from the non-rotating sphere.

**Figure 7.**
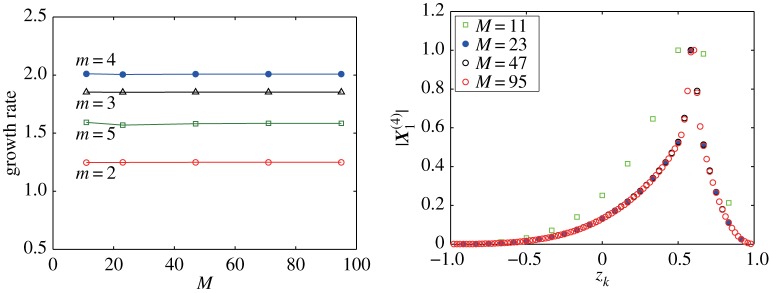
Convergence of the unstable eigenvalues $\sigma_{1}^{\left(m\right)}$ and the eigenvector to the most unstable eigenvalue with respect to *M*. The boundaries of the vortex strip are located at *z*=1/2 and *z*=2/3. The angular speed of the solid body rotation is *Ω*=0.5. The vorticity constants are given by *ω*_*N*_=*ω*_*S*_=1. (*a*) Largest growth rates for the unstable modes 2≤*m*≤5. (*b*) Distribution of the components of the eigenvectors $\left|X_{1}^{\left(4\right)}\right|$ associated with the most unstable eigenvalue $\sigma_{1}^{\left(4\right)}$.

We check the convergence of the eigenvalues and their corresponding eigenvectors with respect to *M*. Figure 7*a* is the plot of the growth rates of the unstable eigenvalue $\sigma_{1}^{\left(m\right)}$, 2≤*m*≤5 for *M*=11, 23, 47, 71 and 95. The vortex strip is set in 1/2≤*z*≤2/3, which corresponds to *k*_*b*_1__=16 and *k*_*b*_2__=24 for *M*=95 for instance. The angular speed of the rotation *Ω*=0.5 is fixed. We find that the growth rate of the eigenvalue $\sigma_{1}^{\left(m\right)}$ for each *m* stays at the same level, which demonstrates a clear convergence of the eigenvalues with *M*. The limit of the eigenvalue is then defined as $\sigma_{\infty }^{\left(m\right)}=\lim_{M\to \infty }\sigma_{1}^{\left(m\right)}$. Figure 7*b* shows the distributions of the components of the eigenvector $X_{1}^{\left(4\right)}$ associated with the most unstable eigenvalue $\sigma_{1}^{\left(4\right)}$ for various *M*. All distributions in figure 7*b* are almost overlapped for *M*≥23, while the eigenvector for *M*=11 differs from the other distributions due to low accuracy. This indicates that the distribution converges to a complex-valued continuous function on *z*∈(−1,1), say $\phi_{\infty }^{\left(4\right)}(z)=\xi_{\infty }^{\left(4\right)}(z)+i\eta_{\infty }^{\left(4\right)}(z)$, with a peak value at the middle of the vortex strip between *z*=1/2 and 2/3. Hence, in the limit as M→∞, this function becomes the eigenfunction associated with the eigenvalue $\sigma_{\infty }^{\left(4\right)}$ for the vortex strip on the rotating sphere with the continuous vorticity distribution. For the other *m*, the same convergence is observed, which similarly yields the eigenfunction $\phi_{\infty }^{\left(m\right)}=\xi_{\infty }^{\left(m\right)}+i\eta_{\infty }^{\left(m\right)}$ to the unstable eigenvalue $\sigma_{\infty }^{\left(m\right)}$ for each *m*. The eigenfunction gives rise to the most unstable flow profile on the rotating sphere and it is represented in three-dimensional Cartesian coordinates by

$$\ell_{\infty }^{\left(m\right)}(z,\alpha )=(\sqrt{1-{\left(z_{\infty }^{\left(m\right)}(z,\alpha )\right)}^{2}}\cos m\alpha ,\sqrt{1-{\left(z_{\infty }^{\left(m\right)}(z,\alpha )\right)}^{2}}\;\sin m\alpha ,z_{\infty }^{\left(m\right)}(\alpha )),$$

where $z_{\infty }^{\left(m\right)}$ is the continuous limit of (3.1):

$$z_{\infty }^{\left(m\right)}(z,\alpha )=1-2z+\xi_{\infty }^{\left(m\right)}(z)\;\cos m\alpha -\eta_{\infty }^{\left(m\right)}(z)\;\sin m\alpha ,\;(z,\alpha )\in [-1,1]\times [0,2\pi ).$$

Let us comment again on the choice of the number of discretizations *M* used in the stability analysis and following numerical computations. Although the result of *M*=23 fairly resolves the distribution of the components of the eigenvector with respect to the profile with the peak, it does not provide good accuracy for other properties of the flow such as the stability diagram. The main reason for this is because of the inaccuracy in the velocity field as we see in figure 2.

**Figure 8.**
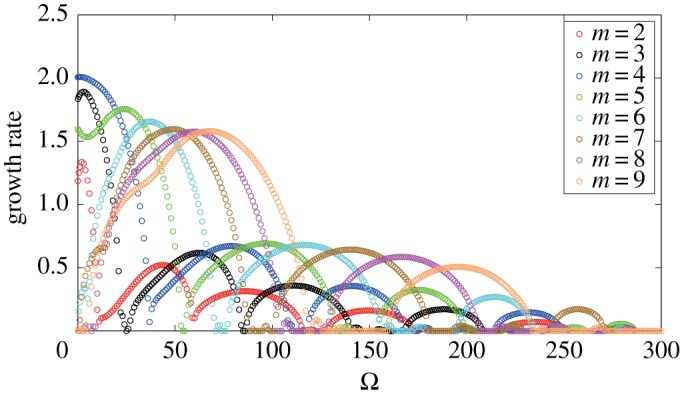
Growth rates of the unstable eigenvalues $\sigma_{1}^{\left(m\right)}$ for 2≤*m*≤9 with respect to the angular speed *Ω* for the vortex strip between *z*_16_=2/3 and *z*_24_=1/2. The vorticity constants are given by *ω*_*N*_=*ω*_*S*_=1.

We now observe the effect of the angular speed of the solid body rotation *Ω* on the eigenvalues and the eigenvectors. Figure 8 shows the growth rates of the unstable eigenvalues $\sigma_{1}^{\left(m\right)}$ for 2≤*m*≤9 with respect to *Ω*, when the boundaries of the vortex strip are placed at *z*_16_=2/3 and *z*_24_=1/2. The order of the growth rates is changed and high modes tend to be the most unstable for larger *Ω*, which is consistent with what we have observed in the stability diagrams of figure 3. By the way, the magnitude of the largest growth rate $\sigma_{1}^{\left(m\right)}$ for all *m* decreases with *Ω*, exhibiting an intermittent behaviour, and becomes zero when *Ω* exceeds 290. This indicates that the flow tends to be stable for large *Ω*, although such a fast rotation would be unphysical, in the geophysical sense. On the other hand, as Ω→0, the growth rates converge to certain finite values. We have checked that these values agree with the non-rotating case [14].

**Figure 9.**
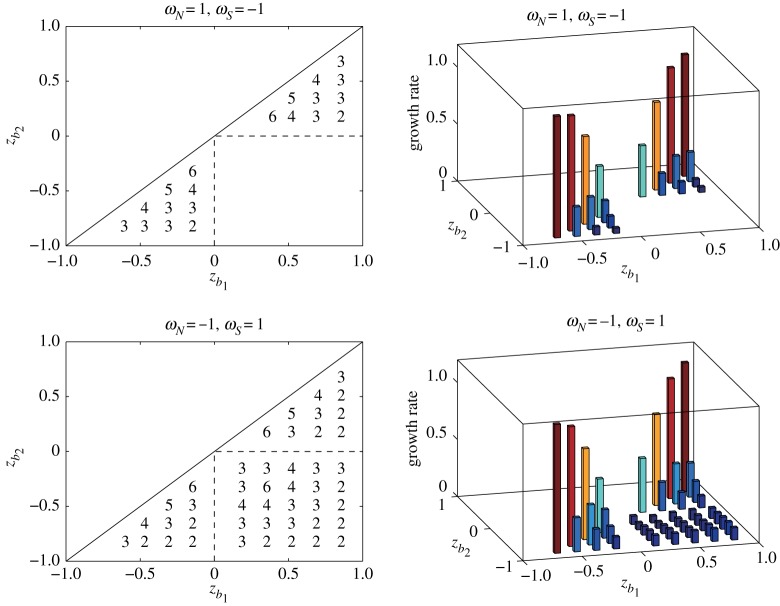
Stability diagrams of the most unstable modes *m*_*max*_ and their corresponding growth rates for different signs of *ω*_*N*_ and *ω*_*S*_. The vorticity constants are taken as (*ω*_*N*_,*ω*_*S*_)=(1,−1) and (*ω*_*N*_,*ω*_*S*_)=(−1,1). The angular speed is *Ω*=0.5, and the number of the discretizing band boundaries is *M*=95.

Finally, we take the vorticity constants *ω*_*N*_ and *ω*_*S*_ as (*ω*_*N*_,*ω*_*S*_)=(1,−1) and (−1,1). Figure 9 shows the stability diagram of the most unstable modes for the two cases. The angular speed of the rotating sphere is set to *Ω*=0.5 and the number of band boundaries is fixed at *M*=95. According to the argument in §2a, for *ω*_*N*_=1 and *ω*_*S*_=−1, the unstable region is split into three regions separated by the line *z*_48_=0, and there is no unstable region in the middle. We find the monotone behaviour of the most unstable mode along a fixed *z*_*b*_1__. For *ω*_*N*_=−1 and *ω*_*S*_=1, all the cases considered for the regions are unstable and the monotone behaviour holds for the upper and lower left regions. The growth rates corresponding to the most unstable modes are also plotted next to the stability diagrams in figure 9. The maximum growth rates are attained when the vortex strip is located in the vicinity of the north and south poles.

**Figure 10.**
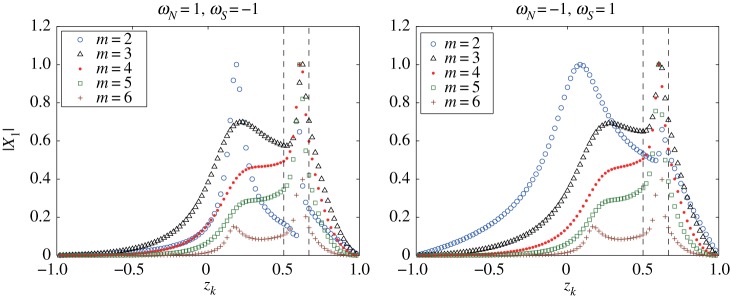
Distributions of the components of the eigenvectors $\left|X_{1}^{\left(m\right)}\right|$ associated with the unstable eigenvalues $\sigma_{1}^{\left(m\right)}$ for 2≤*m*≤6 for the same pairs of *ω*_*N*_ and *ω*_*S*_ corresponding to figure 9. The vortex strip is placed between *z*_16_=2/3 and *z*_24_=1/2.

Figure 10 shows the plots of the distribution of the magnitude of the eigenvectors $\left|X_{1}^{\left(m\right)}\right|$ associated with the unstable eigenvalues $\sigma_{1}^{\left(m\right)}$ for 2≤*m*≤6 corresponding to the two cases of *ω*_*N*_ and *ω*_*S*_ in figure 9, in which the vortex strip is embedded between *z*_16_=2/3 and *z*_24_=1/2, and the most unstable mode is *m*_*max*_=4. In figure 10, the eigenvalue profiles for *m*_*max*_=4 have a peak in the middle of the upper and lower boundaries of the vortex strip for both cases. Let us take a closer look at the eigenvector of the most unstable mode. The value at the lower strip boundary *z*_24_ is smaller than the value at the upper boundary *z*_16_. The components of the eigenvector between *z*_24_ and *z*_36_ have similar magnitudes and, thus they form an unstable band region. An interesting behaviour can, however, be found in the profile of the eigenvectors for *ω*_*N*_=1 and *ω*_*S*_=−1. The distributions for the modes *m*=2 and 3 have strong peaks near the equator. This suggests a possibility that the flow above the equator, not in the middle of the vortex strip, becomes the most unstable, which is not observed for *ω*_*N*_=*ω*_*S*_=1. The distributions of the eigenvector for *ω*_*N*_=−1 and *ω*_*S*_=1 also exhibit a similar behaviour.

## 4. Nonlinear evolutions of the vortex strip

We conduct numerical computations for the nonlinear evolutions of the vortex strip by solving the contour dynamics equation (2.7). We then compare the nonlinear evolutions with the linear stability analysis presented in the previous section. In all results of this section, we approximate the contour vorticity distribution corresponding to the solid body rotation with *M*=95 band boundaries. We set the angular speed at *Ω*=0.5. The initial configuration of the band boundaries is given by

4.1 $$\ell_{k}(\alpha ,0)=(\sqrt{1-z_{k}^{2}(\alpha )}\cos\alpha ,\sqrt{1-z_{k}^{2}(\alpha )}\sin\alpha ,z_{k}(\alpha )),\;\alpha \in [0,2\pi ),$$

for *k*=1,…,*M*, where

4.2 $$z_{k}(\alpha )=1-\frac{2k}{M+1}+0.01\cos m\alpha ,$$

for a given mode *m*. Note that it corresponds to $\zeta_{k}(\alpha ,0)=0.01\cos m\alpha$ in (3.1), which does not coincide with the unstable eigenvectors discussed in the stability analysis. Each boundary is discretized by *N*=512 points and the temporal integration is carried out by using the fourth-order Runge–Kutta method with the time step *Δt*=0.005 or 0.01. The derivative of the boundary with respect to *α* in the integral of (2.7) is calculated through its Fourier series representation in three-dimensional Cartesian coordinates. To compare the nonlinear evolution with the linear stability analysis, let us consider the *p*th Fourier coefficient ${\hat{\zeta }}_{k}^{\left(p\right)}(t)$ of the perturbation,

4.3 $$\zeta_{k}(\alpha ,t)=\sum_{p=-N/2}^{N/2-1}{\hat{\zeta }}_{k}^{\left(p\right)}(t)\;{\text{e}}^{ip\alpha },$$

where *ζ*_*k*_(*α*,*t*) denotes the perturbation added to the *z*-component of the *k*th boundary *z*_*k*_(*α*,*t*) in equation (3.1). We focus on the Fourier amplitudes of mode *p*, which is given by

$$|{\hat{\zeta }}_{k}^{\left(\pm p\right)}(t)|:=|{\hat{\zeta }}_{k}^{\left(p\right)}(t)|+|{\hat{\zeta }}_{k}^{\left(-p\right)}(t)|.$$

**Figure 11.**
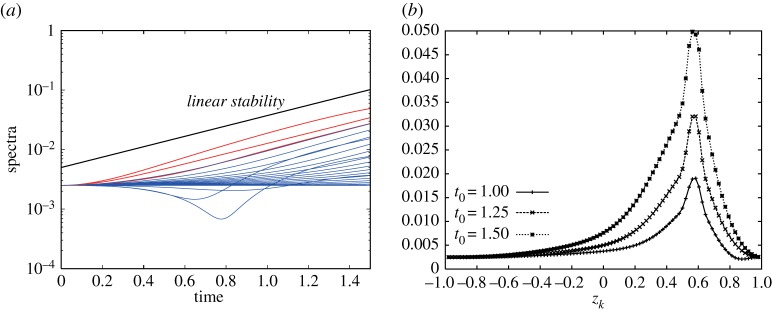
(*a*) Evolution of the Fourier amplitude of the perturbation $\left|{\hat{\zeta }}_{k}^{\left(\pm 4\right)}(t)\right|$ of the band boundaries for 0≤*t*≤1.5. The spectra of every four indices, i.e. *k*=4,8,…92, are plotted. The red curves represent the spectra for the contours in the vortex strip, i.e. *k*=16,20,24, and the blue curves represent the spectra for the others. The vortex strip is placed between *z*_16_=2/3 and *z*_24_=1/2 with the initial perturbation of the most unstable mode *m*_*max*_=4. The solid line represents the exponential growth rate predicted by the linear analysis. (*b*) Snapshots of $\left|{\hat{\zeta }}_{k}^{\left(\pm 4\right)}(t_{0})\right|$ with respect to *z*_*k*_ at *t*_0_=1, 1.25 and 1.5. The vorticity constants are given by *ω*_*N*_=1 and *ω*_*S*_=1. The angular velocity of the rotating sphere is *Ω*=0.5.

**Figure 12.**
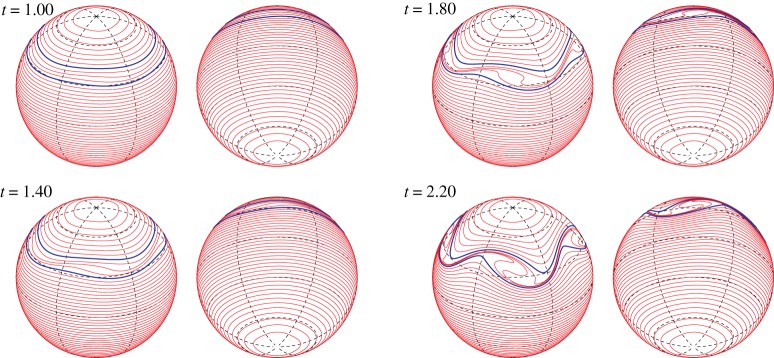
Nonlinear evolution of the vortex strip between *z*_16_=2/3 and *z*_24_=1/2 for the initial perturbation of the most unstable mode *m*_*max*_=4. The vorticity constants are *ω*_*N*_=1 and *ω*_*S*_=1. The angular speed of the rotating sphere is *Ω*=0.5. Each panel shows every two contours viewed from the north pole (right) and the south pole (left).

**Figure 13.**
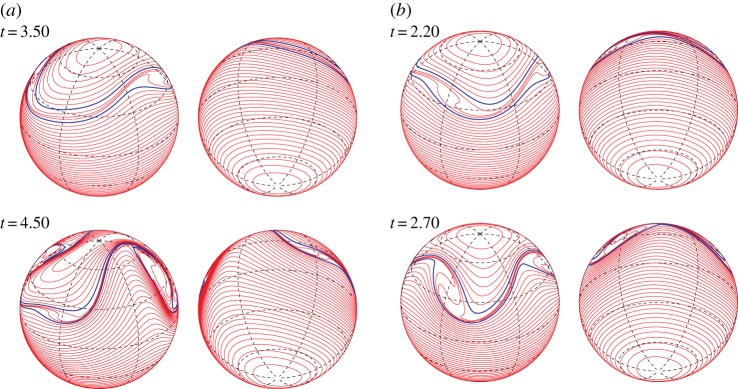
Nonlinear evolutions of the vortex strip between *z*_16_=2/3 and *z*_24_=1/2 for the initial perturbations of the modes (*a*) *m*=2 and (*b*) *m*=3. The vorticity constants are *ω*_*N*_=1 and *ω*_*S*_=1. The angular speed of the rotating sphere is *Ω*=0.5.

We here assume that the vorticity constants are fixed as *ω*_*N*_=1 and *ω*_*S*_=1 and then we change the location of the vortex strip and the mode *m* of the initial perturbation. We first take the boundaries of the vortex strip to be *z*_16_=2/3 and *z*_24_=1/2. Figure 11*a* shows the early time evolution of the Fourier amplitudes $\left|{\hat{\zeta }}_{k}^{\left(\pm 4\right)}(t)\right|$ for the most unstable mode *m*_*max*_=4. Most of the Fourier amplitudes grow exponentially with the same rate predicted by the linear stability analysis. Figure 11*b* gives the snapshots of $\left|{\hat{\zeta }}_{k}^{\left(\pm 4\right)}(t_{0})\right|$ with respect to *z*_*k*_ at times *t*_0_=1, 1.25 and 1.5, in which the subscript 0 means a fixed time hereafter. It shows that the band boundaries in the middle of the vortex strip grow the fastest. The profiles of the snapshots are similar to the eigenvector $\left|X_{1}^{\left(4\right)}\right|$ for *m*_*max*_=4 in figure 5*a*. Therefore, the nonlinear evolution at an early stage is in good agreement with the linear stability analysis. Figure 12 shows the evolution of the band boundaries from *t*=1 to 2.2, where the two boundaries of the zonal strip are shown in blue, while the other band boundaries are shown in red. In figure 12, each panel shows every two contours viewed from the north pole (right) and the south pole (left). After *t*=1.4, the boundaries in the vortex strip deform largely and evolve into a structure with rolling-up vortex cores. The number of the vortex cores is the same as the mode of the initial perturbation *m*_*max*_=4. When we choose the other initial modes *m*=2 and 3, we observe the similar results as shown in figure 13. The exponential growth rates of the Fourier amplitude for both the cases are similar to those given by the linear stability analysis, although they are not shown here. The band boundaries in the middle of the vortex strip become unstable and develop into large rolling-up vortex cores in the strip. The number of the vortex cores again coincides with that of the initial mode.

**Figure 14.**
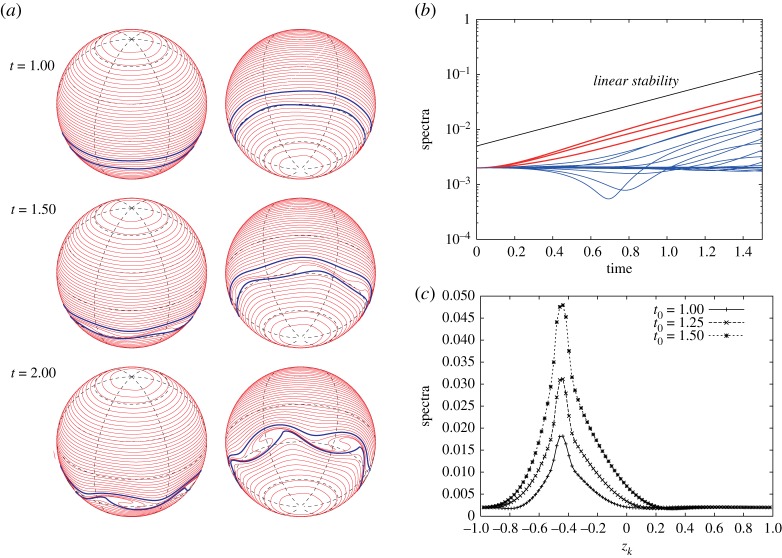
(*a*) Nonlinear evolution of the vortex strip between *z*_64_=−1/3 and *z*_72_=−1/2 for the initial perturbation of the most unstable mode *m*_*max*_=5. The vorticity constants are given by *ω*_*N*_=1 and *ω*_*S*_=1. (*b*) Evolutions of $\left|{\hat{\zeta }}_{k}^{\left(\pm 5\right)}(t)\right|$ for 0≤*t*≤1.5. The spectra of every four indices, i.e. *k*=4,8,…,92, are plotted. The red curves represent the spectra for the contours in the vortex strip, i.e. *k*=64,68,72, and the blue curves represent the spectra for the others. (*c*) Snapshots of $\left|{\hat{\zeta }}_{k}^{\left(\pm 5\right)}(t_{0})\right|$ with respect to *z*_*k*_ at *t*_0_=1, 1.25 and 1.5.

Now we consider the case when the vortex strip is located in the southern hemisphere between *z*_64_=−1/3 and *z*_72_=−1/2. In this case, the width of the vortex strip is unchanged. The most unstable mode for this case is *m*_*max*_=5 as shown in figure 5*a*. Figure 14*a* shows that the band boundaries in the middle of the vortex strip become unstable and they evolve into a structure with five rolling-up vortex cores. The evolution and the snapshots of the Fourier amplitudes at the early stage are also plotted in figure 14*b*,*c*, respectively. The exponential growth rates of the spectra are almost the same as the linear growth rate and the snapshots $\left|{\hat{\zeta }}_{k}^{\left(\pm 5\right)}(t_{0})\right|$ have the peak in the middle of the vortex strip, whose profiles are also similar to the eigenvector $\left|X_{1}^{\left(5\right)}\right|$ for *m*_*max*_=5 in figure 5*d*.

**Figure 15.**
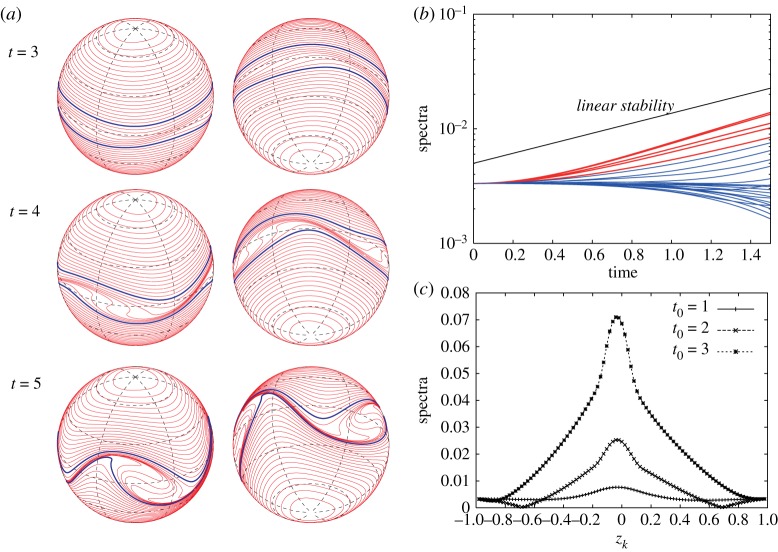
(*a*) Nonlinear evolution of the vortex strip between *z*_40_=1/6 and *z*_56_= −1/6 for the initial perturbation of the most unstable mode *m*_*max*_=3. The vorticity constants are given by *ω*_*N*_=1 and *ω*_*S*_=1. (*b*) Evolution of $\left|{\hat{\zeta }}_{k}^{\left(\pm 3\right)}(t)\right|$ for 0≤*t*≤1.5. The spectra of every four indices, i.e. *k*=4,8,…,92, are plotted. The red curves represent the spectra for the contours in the vortex strip, i.e. *k*=40,44,…,56, and the blue curves represent the spectra for the others. (*c*) Snapshots of $\left|{\hat{\zeta }}_{k}^{\left(\pm 3\right)}(t_{0})\right|$ with respect to *z*_*k*_ at *t*_0_=1, 2 and 3.

**Figure 16.**
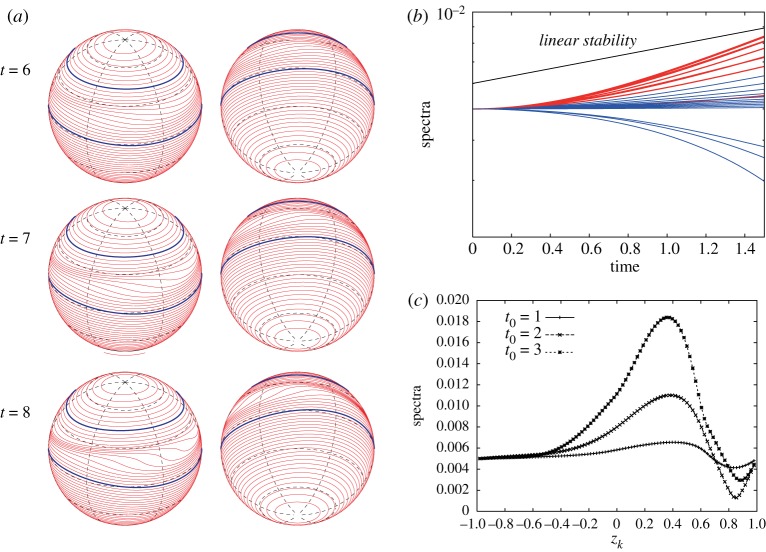
(*a*) Nonlinear evolution of the vortex strip between *z*_16_=2/3 and *z*_48_=0 for the initial perturbation of the most unstable mode *m*_*max*_=2. The vorticity constants are given by *ω*_*N*_=1 and *ω*_*S*_=1. (*b*) Evolution of $\left|{\hat{\zeta }}_{k}^{\left(\pm 2\right)}(t)\right|$ for 0≤*t*≤1.5. The spectra of every four indices, i.e. *k*=4,8,…,92, are plotted. The red curves represent the spectra for the contours in the vortex strip, i.e. *k*=16,20,…,48, and the blue curves represent the spectra for the others. (*c*) Snapshots of $\left|{\hat{\zeta }}_{k}^{\left(\pm 2\right)}(t_{0})\right|$ with respect to *z*_*k*_ at *t*_0_=1, 2, 3.

Let us consider vortex strips with larger widths when the exponential growth rate is small as in figure 4*a*. When we consider the vortex strip between *z*_40_=1/6 and *z*_56_=−1/6 for the initial perturbation of the most unstable mode *m*_*max*_=3 as shown in figure 15, the exponential growth rate of the Fourier amplitudes still agrees with the linear stability analysis. Their snapshots $\left|{\hat{\zeta }}_{k}^{\left(\pm 3\right)}(t_{0})\right|$ at *t*_0_=1, 2 and 3 in figure 15*c* show that the peak location is very near the equator, which is consistent with the eigenvector $\left|X_{1}^{\left(3\right)}\right|$ in figure 5*c*. After the long-time evolution of the zonal jet, the band boundaries in the middle of the vortex strip form three large rolling-up vortex cores at *t*=5. We can however find an inconsistency between the linear stability analysis and the nonlinear evolution when we consider the vortex strip with a larger width. The nonlinear evolution of the vortex strip between *z*_16_=2/3 and the equator *z*_48_=0, and the growth of the Fourier amplitudes for the initial perturbation of the most unstable mode *m*_*max*_=2 are shown in figure 16. We observe a large deformation of the band boundaries in the middle of the vortex strip as in figure 16*a*, but no rolling-up vortex cores appear by *t*=8. Figure 16*b* demonstrates a deviation of the growth rates of the Fourier amplitudes $\left|{\hat{\zeta }}_{k}^{\left(\pm 2\right)}(t)\right|$ from that of the linear stability analysis, and the profiles of $\left|{\hat{\zeta }}_{k}^{\left(\pm 2\right)}(t_{0})\right|$ at *t*_0_=1, 2 and 3 in figure 16*c* are not similar to that of the eigenvectors $\left|X_{1}^{\left(2\right)}\right|$ in figure 5*b*. Nonetheless, the band boundaries in the middle of the vortex strip is the most unstable. In this case, the growth rate of the unstable eigenvalue is very small and it takes a much longer time to evolve. As a result, the linear growth rate does not dominantly drive the evolution of the flow and some nonlinear effects are excited.

**Figure 17.**
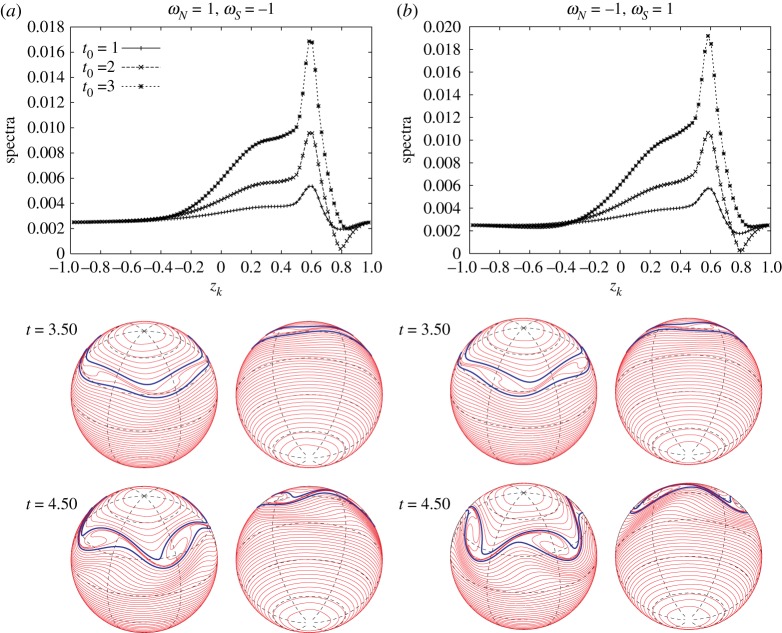
Snapshots of $\left|{\hat{\zeta }}_{k}^{\left(\pm 4\right)}(t_{0})\right|$ with respect to *z*_*k*_ at *t*_0_=1, 2, 3 and the nonlinear evolutions of the vortex strip between *z*_16_=2/3 and *z*_24_=1/2 on the rotating sphere with *Ω*=0.5. The initial perturbation is the most unstable mode *m*_*max*_=4. The vorticity constants are given by (*a*) (*ω*_*N*_,*ω*_*S*_)=(1,−1) and (*b*) (*ω*_*N*_,*ω*_*S*_)=(−1,1).

We now change the signs of the vorticity constants *ω*_*N*_ and *ω*_*S*_, fixing the two boundaries of the vortex strip at $z_{16}=\frac{2}{3}$ and $z_{24}=\frac{1}{2}$. We find more complex behaviours in this case. Figure 17 shows the snapshots of the Fourier amplitudes and the nonlinear evolutions of the vortex strip for the initial perturbation of the most unstable mode *m*_*max*_=4 when the vortex constants are given by (*ω*_*N*_,*ω*_*S*_)=(1,−1) and (*ω*_*N*_,*ω*_*S*_)=(−1,1). In both cases, the growth rates of the Fourier amplitudes agree with the exponential growth rate given by the linear stability analysis, and the snapshots $\left|{\hat{\zeta }}_{k}^{\left(4\right)}(t_{0})\right|$ at *t*_0_=1, 2, 3 are similar to the eigenvectors $\left|X_{1}^{\left(4\right)}\right|$ for the most unstable mode shown in figure 10; there exist a peak in the middle of the vortex strip and an unstable band region between *z*_24_ and *z*_36_. Regarding the nonlinear evolutions, both of the vortex strips develop into four rolling-up vortex cores with opposite rolling-up directions, which is because of the difference in the sign of the vorticity constants.

**Figure 18.**
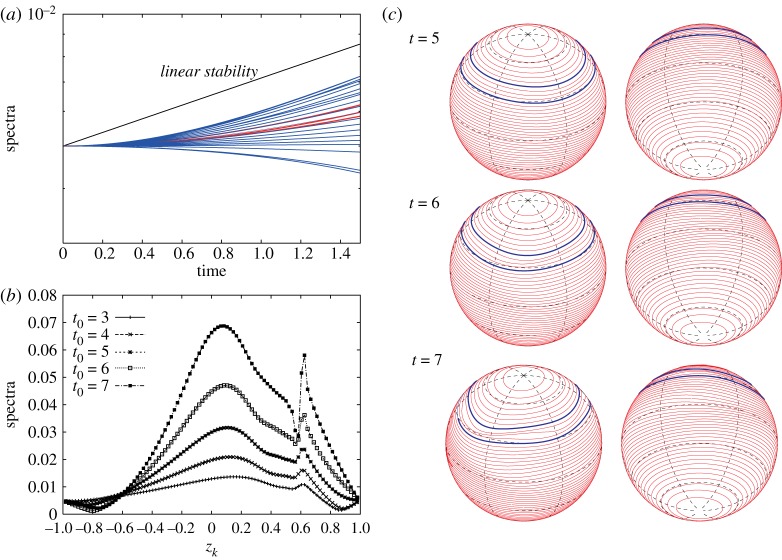
(*a*) Evolution of $\left|{\hat{\zeta }}_{k}^{\left(\pm 2\right)}(t)\right|$ for 0≤*t*≤2. The spectra of every four indices, i.e. *k*=4,8,…,92, are plotted. The red curves represent the spectra for the contours in the vortex strip, i.e. *k*=16,20,24, and the blue curves represent the spectra for the others. (*b*) Snapshots of $\left|{\hat{\zeta }}_{k}^{\left(\pm 2\right)}(t_{0})\right|$ with respect to *z*_*k*_ at *t*_0_=3, 4, 5, 6, 7. (*c*) Nonlinear evolution of the vortex strip between *z*_16_=2/3 and *z*_24_=1/2 for the initial perturbation of mode *m*=2. The vorticity constants are given by *ω*_*N*_=−1 and *ω*_*S*_=1.

We finally show another interesting nonlinear evolution for the initial perturbation of the mode *m*=2 for the vorticity constants *ω*_*N*_=−1 and *ω*_*S*_=1. Figure 18*a* shows the evolution of the Fourier amplitudes $\left|{\hat{\zeta }}_{k}^{\left(\pm 2\right)}(t)\right|$ for 0≤*t*≤2, which indicates that all perturbations grow exponentially at the same growth rate predicted by the linear stability analysis. The snapshots of $\left|{\hat{\zeta }}_{k}^{\left(\pm 2\right)}(t_{0})\right|$ at *t*_0_=3, 4, 5 in figure 18*b* look similar to the eigenvector $\left|X_{1}^{\left(2\right)}\right|$ for *m*=2 in figure 10, which has two peaks above the equator and in the middle of the vortex strip. Hence, in the linear stage, the band boundaries away from the zonal vortex band grow faster. In figure 18*c*, we hardly see a remarkable deformation around the equator at a late time, while there appears a large deformation in the middle of the zonal vortex band. However, the snapshots of the Fourier amplitudes at *t*_0_=6, 7 in figure 18*b* still show that the band boundaries near the equator are growing largely at this stage. This is because the deformation of the mode *m*=2 results in the change of the band boundaries to elliptic curves, which cannot be identified clearly when plotted on the sphere. The result of figure 10 demonstrates that the band boundaries, above the equator, away from the vortex strip may deform largely.

Let us make a technical remark regarding the rotation speed. In the numerical computations, we have considered only one rotation speed *Ω*=0.5. In fact, if we take a sufficiently large rotation speed, the computation is not sustainable for a long time. We have found that when the rotation speed is high, the boundaries do not stay on the sphere and tend to deviate from the surface of the sphere, because they are represented in three-dimensional Cartesian coordinates in the vortex contour dynamics model. This causes the breakdown of the computation at a late time. To overcome this problem, a different mathematical formulation for the equations in the spherical coordinate system is required [28]. Another possible method is to map the zonal band boundaries, approximating the solid body rotation of the sphere, to circular concentric boundaries in a plane by using a conformal stereographic projection as in [27]. We leave these works for future studies.

## 5. Geophysical relevance to planetary jet streams

We examine the geophysical relevance of the model for a vortex strip on a rotating sphere to jet streams in planets. The parameters are estimated using the Rossby number *R*_*o*_=*U*/2*ΩL*, which is a non-dimensional parameter with *Ω*, *L* and *U* being the angular speed, the radius of a planet and a representative latitudinal wind speed in the jet stream, respectively. The planetary parameters of Jupiter, Saturn and Earth are found in [2,4,12] and are summarized in table 1. As *Ω*=0.5 and *L*=1 in our model, it follows from *R*_*o*_≡*U*_*J*_/2*Ω*_*J*_*L*_*J*_=*u*_*J*_/2⋅0.5⋅1=*u*_*J*_ that the representative wind speed for Jupiter is estimated as *u*_*J*_=0.005. Similarly, we obtain the representative speed *u*_*S*_=0.005 for Saturn and *u*_*E*_=0.06 for Earth. Note that the stability analysis for Jupiter and Saturn yields the same results owing to *u*_*S*_=*u*_*J*_. In our model, the velocities of the boundaries of the vortex strip at *z*=*z*_*b*_1__ and *z*_*b*_2__ are given by

$$u_{b_{1}}=\omega_{N}\sqrt{\frac{1-z_{b_{1}}}{1+z_{b_{1}}}}+\Omega \sqrt{1-z_{b_{1}}^{2}}\;\mathrm{and}\;u_{b_{2}}=-\omega_{S}\sqrt{\frac{1+z_{b_{2}}}{1-z_{b_{2}}}}+\Omega \sqrt{1-z_{b_{2}}^{2}}.$$

Suppose *ω*_*N*_=±*ω*_*S*_; then we determine the vorticity constant *ω*_*N*_ as follows:

5.1 $$\omega_{N}=\frac{\Delta u-\Omega (\sqrt{1-z_{b_{1}}^{2}}-\sqrt{1-z_{b_{2}}^{2}})}{\sqrt{\left(1-z_{b_{1}})/(1+z_{b_{1}}\right)}\pm \sqrt{\left(1+z_{b_{2}})/(1-z_{b_{2}}\right)}},$$

where *Δu*=*u*_*b*_1__−*u*_*b*_2__ is equivalent to the representative wind speed defined above.

**Table 1.** Planetary parameters of Jupiter, Saturn and Earth used.

	rotation speed (s^−1^)	radius (m)	wind speed (*m* *s*^−1^)
Jupiter	*Ω*_*J*_=1.74×10^−4^	*L*_*J*_=7.0×10^7^	*U*_*J*_=140
Saturn	*Ω*_*S*_=1.64×10^−4^	*L*_*S*_=6.0×10^7^	*U*_*S*_=100
Earth	*Ω*_*E*_=7.30×10^−5^	*L*_*E*_=6.4×10^6^	*U*_*E*_=55

**Figure 19.**
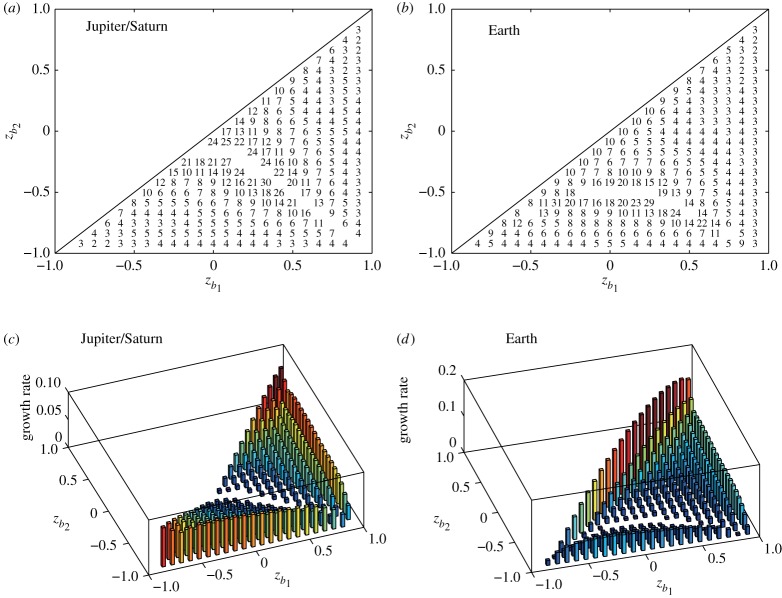
The stability diagrams for the parameters relevant to (*a*) Jupiter/Saturn and (*b*) Earth. Under the assumption *ω*_*N*_=*ω*_*S*_ and *Ω*=0.5, the polar vorticity constants are determined from (5.1) with *u*_*J*_=*u*_*S*_=0.005 and *u*_*E*_=0.06, respectively. The growth rates of the most unstable mode for the cases of (*c*) Jupiter/Saturn and (*d*) Earth are plotted.

Figure 19 is the stability diagrams for the most unstable modes and their corresponding growth rates for the parameters relevant to Jupiter/Saturn and Earth. The continuous vorticity distribution corresponding to the solid body rotation is approximated by *M*=95 band boundaries. The rotational speed is *Ω*=0.5 and we assume *ω*_*N*_=*ω*_*S*_. For the vortex strip between *z*_*b*_1__ and *z*_*b*_2__ with *b*_1_=4*p* and *b*_2_=4*q* (1≤*p*<*q*<23), the value of the vorticity constant *ω*_*N*_ is obtained from (5.1) for *Δu*=*u*_*J*_=*u*_*S*_=0.005 (Jupiter/Saturn) and *Δu*=*u*_*E*_=0.06 (Earth).

In figure 19, we first pay attention to the stability of vortex strips with small width, i.e. for *b*_1_=4*p* and *b*_2_=4(*p*+1), which are aligned just below the diagonal line of the stability diagrams, because those localized jet structures are often observed in many planetary atmospheric flows. In Jupiter/Saturn case, the most unstable modes for polar vortex strips tend to be low with large growth rate, whereas those for equatorial strips become high with small growth rate. On the other hand, in the case of Earth, the most unstable modes for polar vortex strips are lower than those for equatorial strips, which is similar to the case of the Jupiter/Saturn. However, their corresponding growth rates are different: the growth rates for vortex strips in the mid-latitude of the northern hemisphere are larger than those for polar vortex strips. Note that the stability diagrams for *ω*_*N*_=−*ω*_*S*_ are similar to those in figure 19*a*,*b* for 0<*z*_*b*_2__<*z*_*b*_1__<1 and are not shown here. In figure 19*a*, we also find a stable region along the line *z*_*b*_1__=*z*_*b*_2__ for Jupiter/Saturn case, where all perturbations up to *m*=100 wavenumber become neutrally stable. Another stability region appears along the region of *z*_*b*_2__=−0.5 as we see in figure 19*b* for the Earth case. It is not evident whether the presence of such a stable region has a certain geophysical significance, which should be investigated in the future.

We next examine the stability of specific vortex strips corresponding to strong polar jets observed at about 75–80°*N* of Saturn [12]. As the width of vortex strips is so narrow that their boundaries do not coincide with those of the band boundaries for *M*=95, we use a finer approximation *M*=383. As the boundary locations 1≤*b*_1_≤*b*_2_≤*M* are chosen arbitrarily, we have checked the three cases of (*b*_1_,*b*_2_)=(3,5), (4,6) and (6,8) when the localized vortex strips are located in the latitude range 75 and 80°*N*. The stability analysis shows that the vortex strips are unstable for all cases and their corresponding most unstable modes are given by *m*_*max*_=4 for (*b*_1_,*b*_2_)=(3,5), *m*_*max*_=5 for (*b*_1_,*b*_2_)=(4,6) and *m*_*max*_=6 for (*b*_1_,*b*_2_)=(6,8). This means that, for the zonal strip between *z*_6_ and *z*_8_ at 74–76°*N*, the hexagonal perturbation grows the most rapidly. In [12], a hexagonal circumpolar jet stream of Saturn was numerically demonstrated from a general circulation model. Here, we also confirm the hexagonal instability of the polar jet from the barotropic vortex contour dynamics.

## 6. Conclusion

We have studied the motion of the vortex strip on a rotating sphere using the barotropic flow model, approximating the continuous vorticity distribution induced by the effect of the rotating sphere with the zonal bands of uniform vorticity. The main contribution of this work is discovering the stability of the vortex strip subject to the solid body rotation by taking the large number of the band boundaries. We have investigated the linear stability of the vortex strip when the polar vorticity constants have the same magnitude, i.e. *ω*_*N*_=*ω*_*S*_=1. The physical parameters are the angular speed of rotation sphere and the width of the vortex strip. For a standard angular speed *Ω*=0.5, the linear stability analysis shows that the exponential growth rate becomes larger as the width gets smaller, regardless of the locations of the vortex strip. When the narrow vortex strip is located around the equator, it has the largest growth rate. For a small *Ω*≈0, the rotation generates an instability on the wide vortex strips, unlike the non-rotating case. However, as *Ω* increases, the vortex strip tends to be less unstable and the most unstable mode gets higher. For a sufficiently large *Ω*, the vortex strip becomes neutrally stable, which reveals a stabilizing effect owing to the rotation of the sphere.

We have performed the numerical computations of the nonlinear evolution of vortex strips on the rotating sphere. When the width of the vortex strip is small, the exponential growth rate of the Fourier amplitudes in the nonlinear evolutions at early times fits well with that predicted by the linear stability analysis. Furthermore, the profiles of the Fourier amplitudes are similar to the eigenvector corresponding to the unstable eigenvalue, and the band boundaries in the middle of the strip are the most unstable among all band boundaries. Hence, the unstable eigenvector suggests which boundary becomes the most unstable in the nonlinear evolution. After a long-time evolution, the vortex strip develops into a varicose large structure with rolling-up vortex cores, whose number is the same as the mode of the initial perturbation. However, a deviation of the growth rate of the nonlinear evolution from the linear stability analysis is apparent when the width is large. This may be due to small growth rates and excitation of some nonlinear effects.

We have also studied the stability of the vortex strip when the polar vorticity constants have opposite signs, (*ω*_*N*_,*ω*_*S*_)=(1,−1) and (*ω*_*N*_,*ω*_*S*_)=(−1,1). The linear stability analysis shows that the exponential growth rate corresponding to the most unstable eigenvalue becomes larger as the width is smaller; however, the most unstable location of the vortex strip is in the polar regions, and not around the equator. The eigenvector similarly provides information which band boundary becomes the most unstable, but it has different and complex profiles from those for *ω*_*N*_=*ω*_*S*_=1, where there is a peak outside of the vortex strip and an unstable band region. In the nonlinear evolution, the vortex strip of a small width in the northern hemisphere evolves into a structure of rolling-up vortex cores in both cases; however, the rolling-up direction for (*ω*_*N*_,*ω*_*S*_)=(1,−1) is opposite to that for (*ω*_*N*_,*ω*_*S*_)=(−1,1). Importantly, boundaries far away from the vortex strip can be unstable, for a different choice of mode of the initial perturbation. This phenomenon suggests that the polar jet stream yields an instability of atmospheric flows around the equator under certain circumstances. We conclude that the solid body rotation gives significant influences on the stability and the evolution of the flow of the vortex strip.

We have examined the geophysical relevance of the model for a vortex strip to the planetary jet streams such as Jupiter, Saturn and Earth. We find that the stability diagram and growth rates of Jupiter and Saturn are very different from those of Earth. For Jupiter and Saturn, the polar vortex strips have the largest growth rate, while for Earth, the strips in the mid-latitude of the northern hemisphere are the largest. This behaviour might be related to appearance of the jet stream near the north pole for Saturn and in the mid-latitude for Earth. Furthermore, the linear stability analysis for Saturn demonstrates the hexagonal instability for the vortex strip at 74–76°*N*, which is in a reasonable agreement with the observation of the polar jet stream [1]. Therefore, our simple model based on vortex contour dynamics not only describes qualitative behaviours but also provides quantitative prediction for the stability of jet streams.

## Data accessibility

This article has no additional data.
